# The First Case of Ignatzschineria ureiclastica/larvae in the United States Presenting as a Myiatic Wound Infection

**DOI:** 10.7759/cureus.14518

**Published:** 2021-04-16

**Authors:** Kasey Reed, Samuel B Reynolds, Clayton Smith

**Affiliations:** 1 Internal Medicine/Pediatrics, University of Louisville School of Medicine, Louisville, USA; 2 Internal Medicine, University of Louisville School of Medicine, Louisville, USA

**Keywords:** ignatzschineria, myiasis infestation, ignatzschineria ureiclastica, ignatzschineria larvae, bacteremia

## Abstract

*Ignatzschineria* is a recently identified genus of bacteria that has been isolated from the digestive tract of multiple flies associated with decomposing tissue. Species within this genus are rarely implicated in human disease, and less than 10 cases worldwide have been documented in the literature. Although there have been several documented cases of *Ignatzschineria indica* bacteremia in the United States (with one previous case in Louisville, KY), this case represents the first documented case of *Ignatzschineria ureiclastica/larvae* bacteremia in the United States. The natural insect host of this bacteria, parasitic flies that are commonly found among sheep and other livestock, may pose a public health hazard in the city and implicate geographic spread of this bacteria species and its host.

## Introduction

As a bacterial isolate, *Ignatzschineria* is most commonly found in the digestive tract of *Wohlfahrtia magnifica*, which is a parasitic fly that plays a significant role in the development of myiatic infections in livestock [1,2]. The patient in this case lived less than five miles from a large swine slaughterhouse in downtown Louisville, Kentucky. His psychiatric illness and drug abuse prevented him from taking adequate care of his surgical wounds, which were subsequently infected with the larvae of a fly (species unknown). The presence of the slaughterhouse near a large urban population that includes many homeless, mentally ill, and other vulnerable people may pose a previously unrecognized threat to their health and well-being.

## Case presentation

A 66-year-old African American male presented to the emergency department with a left lower extremity wound infection with maggot infestation. Six months prior to this presentation, he had sustained a tibial fracture that required open reduction and internal fixation. His post-operative course over the following four months was complicated by wound dehiscence that required multiple skin grafts to close, and he was intended to be discharged home with vacuum-assisted closure in place. He was unfortunately lost to follow-up two months prior to this recurrent hospital presentation for myiatic infection. His medical history was notable for schizophrenia and cocaine and opioid use disorders, all of which were untreated and contributed to his state of self-neglect that led to his presentation.

On presentation, vital signs were unremarkable with temperature of 98.7 degrees Fahrenheit orally, heart rate of 86 beats per minute, blood pressure of 103/77, and normal respiratory rate and oxygen saturation on room air. Physical examination was only notable for a left lower extremity skin lesion with exposed subcutaneous tissue, brown serosanguinous drainage, foul odor, and presence of maggots on the wound and dressing. Admission laboratory studies were noteworthy only for mild elevations in C-reactive protein (34 mg/L) and leukocytes (13,100 cells/mm^3^). The wound was thoroughly irrigated with normal saline and Betadine®, and all larvae were removed in the emergency department, unfortunately prior to any photographic documentation of the wound. He was admitted initially to orthopedic surgery for the management of post-operative wound infection and was taken to the operating room the following day for thorough wound debridement.

**Figure 1:**
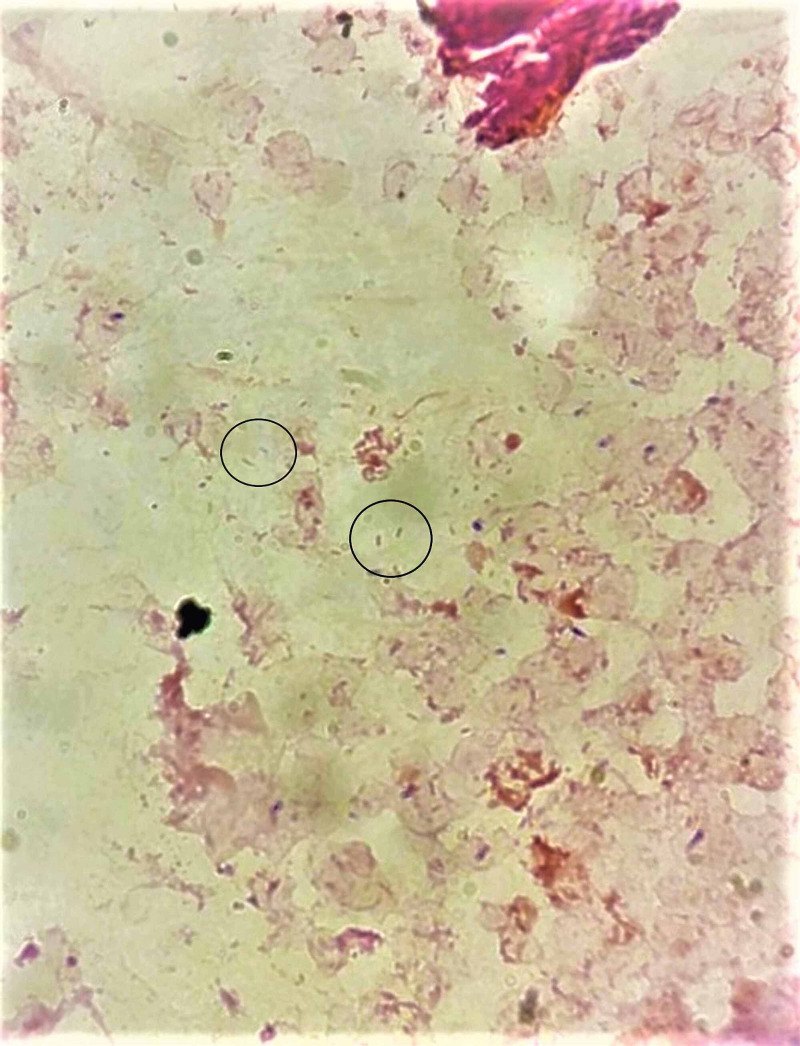
Gram stain of blood culture showing gram-negative rods of Ignatzschineria ureiclastica/larvae.

The following day, two sets of blood cultures obtained from admission were positive for gram-negative bacteria (Figure 1), prompting initiation of piperacillin-tazobactam. Internal medicine service was consulted for assistance in management at this time. Using traditional laboratory PCR (polymerase chain reaction), a bacterium was not initially identified, which prompted 16s ribosomal subunit sequencing and subsequent speciation of *Ignatzschineria ureiclastica* or *I. larvae*, as per report. The bacterium also demonstrated pan-sensitivity to all antibiotics with typical gram-negative coverage, including ampicillin. Repeat blood cultures taken after debridement were positive in one of two sets for extended-spectrum β-lactamase (ESBL)-producing *Klebsiella pneumoniae*, prompting a transition to meropenem for two weeks.

During this hospital course, there were detailed discussions between the infectious disease, orthopedic, and internal medicine services about the potential for polymicrobial osteomyelitis given polymicrobial bacteremia in the setting of chronic soft tissue infection. However, due to down-trending inflammatory markers and a concern about patient adherence and cooperation with the treatment plan, he was not taken to the operating room for bone biopsy and no advanced imaging was done to evaluate for osteomyelitis due to the patient's consistent improvement and poor surgical candidacy with concomitant mental illness.

After approximately one week of inpatient antibiotic therapy and vacuum-assisted wound closure, the patient was approved to continue meropenem for a total course of two weeks to treat bacteremia. A peripherally inserted central catheter (PICC) line was placed just prior to planned discharge to a skilled nursing facility. Unfortunately, the patient eloped a day before his planned discharge, but he returned the following day for PICC line removal. He ultimately did not complete the recommended course of antibiotics or subacute rehabilitation and was subsequently lost to follow-up. He returned only once more to the emergency department approximately six months later for evaluation prior to incarceration, and there was no documentation of further wound infection.

## Discussion

*Ignatzschineria* is a genus of bacteria that was previously known as *Schineria*, which was renamed in 2007 to honor Ignatz Rudolph Schiner [1]. Schiner was the first to describe the fly *Wohlfahrtia magnifica*, also known as the spotted flesh fly, whose larvae are inhabited by *Ignatzschineria *species [3]. Less than 10 cases of human infections with *Ignatzschineria* bacteremia with any species of the genus are reported in the literature worldwide, with all reported cases in the United States being due to *Ignatzschineria indica*. In Europe and Asia, the majority of cases are due to *I. indica*, with only a few attributed to* I. larvae/ureiclastica* [4-9]. Interestingly, there is one other reported case of *Ignatzschineria* bacteremia from Louisville, Kentucky, where this patient resided. However, this case was due to the more common *I. indica* [4,9]. This genus is so rare that the sequencing lab in this case was not able to narrow the species beyond *I. ureiclastica* or *I. larvae*.

The only other documented case of *I. ureiclastica* infecting a human occurred in a patient who was found unconscious in a forest in the Loirs Valley of France [5]. The patient presented in fulminant sepsis and succumbed to the infection despite treatment with broad-spectrum antibiotics. There were no reported cases of confirmed solo *I. larvae* in the literature at the time of writing. Interestingly, the host organism of* Ignatzschineria* (the fly *Wohlfahrtia magnifica*) is endemic to Southeastern Europe, Russia, China, and North Africa [6]. The cases of *Ignatzschineria* bacteremia that have been correlated with a particular species of fly in the United States have been associated with *Phaenicia sericata* [6]. This species of fly is the source of the sterile larvae that are used in "maggot therapy," which indicates that patients who are treated with these larvae may be susceptible to *Ignatzschineria* infections [6].

The presence of *Ignatzschineria* infections in North America, in summary, raises concern that there may be a geographical expansion of the parasitic fly, *Wohlfahrtia magnifica*, or that there is potential for the sterile larvae used in wound debridement to be hosts to these possible pathogens. The presence of two cases of *Ignatzschineria* bacteremia (although with different species) in the same city is even more concerning since infection is so rare in humans. This suggests that there is a source of parasitic flies near a large and vulnerable human population that is contributing to the spread of disease and myiatic infections in humans. In Louisville, Kentucky, the clearest source is the large swine slaughterhouse that is within five miles of both patients who have been infected with *Ignatzschineria* species. Further studies are needed not only to better understand this connection but also to ensure the public health and safety of at-risk patients.

## Conclusions

*Ignatzschineria *is a genus of bacteria that has been recently implicated in human disease. It may be underdiagnosed since it commonly presents in polymicrobial infections. More information is needed about the epidemiology and pathogenesis of this bacteria in order to fully appreciate its role in the lives of humans and animals. As the boundaries between large meat production facilities and urban environments continue to cross paths, physicians should consider the potential for human health impacts that are yet unidentified. The identification of *Ignatzschineria* infections in humans in North America may indicate not only that the bacteria can be found in various *Wohlfahrtia* species but also that the fly *Wohlfahrtia magnifica*, the most significant source of zoonotic myiatic infections in animals worldwide, is spreading. The identification of *I. ureiclastica*/*larvae* infection in North America indicates that the worldwide distribution of this infection is growing, and the prevalence of the infection is likely to follow.
